# 

**Published:** 1908-08-01

**Authors:** 


					! AUG. 10.
i
Hospital
Telegraphic Address ] THE MODERN Telephone Number:
Oepedale. Loadop. jj MEDICAL JOURNAL. 'I Gerrard.
NEW SERIES. No. 73, Vol. III. Qatttp^av
No. 1145. Vol. XLIV. oATUKDAY,
August 1st, 1908.
PRipETHltiiPEHCE
\

				

